# An exploratory study of the efficacy and safety of amenamevir for the treatment of herpes zoster in patients receiving immunosuppressive drugs

**DOI:** 10.1111/1346-8138.17364

**Published:** 2024-07-24

**Authors:** Shinichi Imafuku, Satoshi Takeuchi, Kazunori Urabe, Masataka Arakawa, Ryo Sasaki, Daigo Oka, Takenobu Yamamoto, Fumitake Ono, Shigeho Shirahama, Shinichiro Yasumoto, Hiroaki Fukuda

**Affiliations:** ^1^ Department of Dermatology and Cosmetic Surgery Fukuoka University Hospital Fukuoka Japan; ^2^ Department of Dermatology, Federation of National Public Service Personnel Mutual Aid Associations Hamanomachi Hospital Fukuoka Japan; ^3^ Department of Dermatology and Allergology National Hospital Organization Kyushu Medical Center Fukuoka Japan; ^4^ Department of Dermatology Kurume University Hospital Fukuoka Japan; ^5^ Department of Dermatology Hiroshima Red Cross Hospital and Atomic‐Bomb Survivors Hospital Hiroshima Japan; ^6^ Department of Dermatology Kawasaki Medical School Hospital Okayama Japan; ^7^ Department of Dermatology Kawasaki Medical School General Medical Center Okayama Japan; ^8^ Sasori Dermatology Clinic Gifu Japan; ^9^ Department of Dermatology Seirei Mikatahara General Hospital Shizuoka Japan; ^10^ Yasumoto Dermatology Clinic Fukuoka Japan; ^11^ Medical Affairs, Maruho Co., Ltd Osaka Japan; ^12^ Present address: Department of Dermatology Omuta City Hospital Fukuoka Japan; ^13^ Present address: Oka Dermatology Clinic Okayama Japan

**Keywords:** amenamevir, exploratory study, herpes zoster, immune suppression, immunocompromised patient

## Abstract

Amenamevir is an oral once‐daily antiherpesvirus drug that can be administered without dose adjustment in patients with impaired renal function. There are currently no clinical data on immunocompromised patients with herpes zoster treated with amenamevir. Therefore, an exploratory study of the efficacy and safety of amenamevir against herpes zoster in patients with immunosuppression was conducted. Inclusion criteria included patients with acute herpes zoster receiving immunosuppressive drugs or those with malignant tumors or autoimmune diseases. Twenty‐four patients were included and received amenamevir (400 mg once daily after meals) for up to 14 days. The primary end point of overall improvement in skin symptoms 7 days after treatment initiation (day 7) was 58.3% for “markedly improved” and 20.8% for “improved.” The combined improvement rate was 79.2% (95% confidence interval, 57.8–92.9), and 20.8% of patients experienced “worsened” symptoms. The secondary end points of overall improvement in skin symptoms on day 14 and day 28 were 95.7% and 100%, respectively. The skin symptoms progressed during treatment, peaking on day 7, and then began to heal. By Kaplan–Meier estimation, the median periods to complete crusting and healing were both day 14. There were five adverse events with a possible causal relationship to amenamevir (bacterial skin infection, anemia, hyponatremia, headache, and abnormal liver function) in one of the 24 patients. Although the bacterial skin infection was severe, all events in this patient were reported to be either recovered or recovering. These findings indicate that amenamevir can be effective and safe in immunocompromised patients with herpes zoster. However, as worsening can happen around day 7, it is necessary to carefully monitor such patients and switch to other therapies such as intravenous acyclovir if necessary.

**Clinical trial identifier**: Japan Registry of Clinical Trials jRCTs031190208.

## 1 INTRODUCTION

Varicella zoster virus (VZV) causes varicella as a primary infection and then establishes latency in the sensory nerves throughout life. VZV can be reactivated by aging, fatigue, stress, or immunosuppression caused by drugs and/or malignancy. Approximately 10% to 30% of individuals with a history of varicella infection will develop herpes zoster at least once in their lifetime,^1^, ^2^ and it has been reported that about 50% of those aged 85 years have experienced herpes zoster.^3^ Reactivated VZV produces dermatomally distributed erythema and blisters, often with serious pain.^4^, ^5^

Herpes zoster develops more frequently in immunocompromised patients, such as those with malignancies or autoimmune diseases, than in the general population. Impaired cellular immunity to VZV contributes to the onset of herpes zoster in such patients.^2^, ^6^ According to a survey conducted in the United States between 2005 and 2009, incidence rates of herpes zoster in immunocompromised individuals (including post‐hematopoietic stem cell transplant recipients, organ transplant recipients, patients with HIV infection, patients with systemic lupus erythematosus, patients with rheumatoid arthritis, and patients with cancer) ranged from 11.70 to 43.03 per thousand person‐years, which was more than twice the overall reported incidence rate of 4.82 per 1000 person‐years.^7^

Oral antiherpesvirus drugs are commonly used to treat herpes zoster. However, the nucleic acid analogs acyclovir, valacyclovir, and famciclovir are renally excreted, and, in patients with impaired renal function, the dosage should be adjusted according to the level of impairment.

Amenamevir is a unique helicase and primase inhibitor that is indicated for the treatment of herpes zoster with a once‐daily dose.^8^ Amenamevir is absorbed mainly in the small intestine and is metabolized by cytochrome P450 3A (CYP3A) in the liver; around 75% is excreted in the feces (mainly as metabolites) and 21% in the urine (mainly as parent drug and metabolites). The decreased glomerular filtration rate minimally affects the pharmacokinetics of amenamevir in patients with severe renal dysfunction (creatinine clearance <30 mL/min) or in those undergoing hemodialysis.^9^ Thus, amenamevir can be safely administered to a wide range of patients with renal impairment due to comorbid diseases or their treatment without dose adjustment. These advantageous attributes of amenamevir suggest that it can be widely used to treat herpes zoster in patients with various comorbidities; however, the clinical data on amenamevir treatment in such patients are limited. Therefore, we conducted an exploratory study to assess the efficacy and safety of amenamevir against herpes zoster in patients receiving immunosuppressive drugs. In addition, the relationship between the change in VZV‐specific antibody titer and efficacy was also examined.

## 2 METHODS

This study was conducted in compliance with the ethical principles based on the Declaration of Helsinki (revised in 2013), and all applicable Japanese clinical trial regulations. The research protocol for this study was reviewed and approved by the Okinawa Tokushukai Clinical Research Review Board (CRB3200003) on January 10, 2020. A summary of this study was registered in the Japan Registry of Clinical Trials with the identifier jRCTs031190208.

### 2.1 Study design

This open‐label, uncontrolled, single‐arm, multicenter study was conducted between February 13, 2020, and October 25, 2021, at seven medical institutions in Japan (Table S1).

Amenamevir was administered orally, at a dose of 400 mg once daily after meals, for 7 days. Depending on the patient's skin symptoms, further doses of amenamevir could be given for up to 7 additional days at the investigator's discretion. The study design is shown in Figure S1.

### 2.2 Patients

Study participants were aged 20 years or older, receiving immunosuppressive drugs for the treatment of conditions such as malignant tumors or collagen diseases, or to suppress rejection after organ transplantation, and scheduled to receive amenamevir within 5 days after the appearance of a herpes zoster rash. Patients were excluded if they had a history of hypersensitivity to amenamevir; were receiving rifampicin; were not expected to respond adequately to oral antiherpesvirus drugs; had received antiviral drugs (excluding anti‐influenza or ophthalmic drugs) or immunoglobulin reagents within 14 days prior to obtaining consent; were unable to discontinue everolimus, cyclosporine, sirolimus, or tacrolimus during treatment with amenamevir; were receiving treatment for HIV or acquired immunodeficiency syndrome; were currently pregnant or breastfeeding or planning to become pregnant during the study period; or were otherwise deemed ineligible by the study investigators. All patients provided written consent for study participation.

### 2.3 End points

The primary efficacy end point was the proportion of patients with improvement in skin symptoms on day 7. The secondary efficacy end points were the proportion of patients with improvement in skin symptoms on day 14 and at the end of the observation period, the number of days to resolution, the number of days to complete crusting, the number of days to pain resolution, change in pain score using a numeric rating scale (NRS), and improvement in quality of life (QOL). VZV‐specific antibody titers were also measured. Adverse events (AEs) and laboratory test results were collected to evaluate safety.

### 2.4 Assessments

#### 2.4.1 Evaluation of skin symptoms

The severity of skin symptoms was judged based on the affected area. The percentage of the area of erythema/vesicles within the innervated area on the day amenamevir was first administered (day 1) was used to rate the rash as mild (<30%), moderate (30%–70%), or severe (>70%).

The skin symptoms were evaluated by a Central Evaluation Committee (Table S2). Photographs of the affected area were taken on days 1, 7, 14, and 28; comparison of later photographs with that taken on day 1 allowed symptoms to be categorized as “markedly improved,” “improved,” “slightly improved,” “no change,” “worsened,” or “indeterminate.”

Skin symptoms were evaluated on days 1, 4, 7, 14, and 28, and included items such as the presence/absence of skin eruptions, the type of skin eruption, the presence/absence of complete crusting and healing, and the number of days required for resolution. The types of rash were erythema/papule, bullae/pustules, erosions/ulcers, and crusts. Complete crusting was defined as: (i) disappearance of all erythema/papules, blisters/pustules and erosions/ulcers, and crusting of all skin lesions, or (ii) disappearance of all erythema/papules in the case that blisters or pustules did not form. Resolution was defined as: (i) complete disappearance of erythema/papules, blisters/pustules, and erosions/ulcers, and complete disappearance of crusts or epithelialization of the subcrustal bed, or (ii) disappearance of all erythema/papules in the case that no blisters or pustules were formed.

#### 2.4.2 Other evaluations

Pain and QOL were assessed on days 1, 4, 7, 14, and 28. Pain was rated using an NRS, with 11 levels ranging from 0 (“no pain”) to 10 (“worst imaginable pain”). QOL was assessed using the short‐form 8‐item health survey (SF‐8).

VZV‐specific IgG and IgM antibody titers were measured by an enzyme immunoassay method on days 1 and 7.

AEs were recorded from the start of treatment to the time of the last observation, and categorized using the *Medical Dictionary for Regulatory Activities*, Japanese version 23.0. Hematological, biochemical, and urinalysis tests were performed on days 1 and 7.

### 2.5 Statistical analysis

The purpose of this study was to conduct an exploratory evaluation of amenamevir efficacy and safety in patients who developed herpes zoster while being treated with immunosuppressive drugs. Due to an absence of similar studies, the number of cases was not set based on statistical hypothesis testing. Instead, the target enrollment number was set at 50, in consideration of the feasibility of conducting the study at the participating institutions. Efficacy outcomes were conducted in the full analysis set (FAS; all patients who received at least one dose of amenamevir and had at least one efficacy measurement), and safety was assessed in the safety analysis population (SAP; all patients who received at least one dose of amenamevir and had at least one safety measurement). The per‐protocol set (PPS) included all patients from the FAS without a protocol violation during the study period.

For the primary efficacy end point, frequencies were tabulated for each evaluation category. Cases judged to be either markedly improved or improved were defined as improvement, and the overall improvement percentage and 95% confidence interval (CI) were calculated.

Improvement percentages were calculated at the same manner on day 14 and at the end of the observation period. Changes in skin symptoms were tabulated by each observation and evaluation time point. The 25th, 50th (median), and 75th percentiles of the number of days to resolution were determined using the Kaplan–Meier method, with day 1 as the starting point and each rash resolution point as the event. The number of days to complete crusting, resolution, and pain resolution were similarly determined, with complete crusting or resolution or pain resolution (NRS value: 0) as the respective event. In addition, the change in pain NRS score was determined for each time point after the start of treatment. QOL improvements were assessed using the SF‐8 subscales at each evaluation visit. In addition, a score was calculated for each assessment time point, and summary statistics and the degree of improvement were calculated. For both NRS and SF‐8 scores, statistical differences from day 1 to each evaluation time point were calculated using a paired *t* test.

AEs were tabulated in terms of occurrence status and frequency of AEs and adverse reactions by type.

No adjustments for multiplicity were made in this study. The two‐sided significance level for all tests was 5%. All statistical analyses were performed using SAS software version 9.4 (SAS Institute, Inc.) for Microsoft Windows.

## 3 RESULTS

### 3.1 Patient characteristics

The enrollment period for this study coincided with the epidemic period of the novel coronavirus disease 2019 (COVID‐19), and patient registration was slower than expected. Therefore, analysis was performed on the 24 patients who were enrolled in the study, all of whom were included in both the FAS and SAP. Two patients were excluded from the PPS (Figure S2) after 7 days of amenamevir administration. One patient withdrew consent, and one was found to be ineligible (amenamevir administration was started 6 days after the rash appeared, i.e. not within the 5 days specified in the protocol). In total, 20 patients (83.3%) received amenamevir for 7 days, and four patients (16.7%) received amenamevir for 8 to 14 days. The compliance rate for amenamevir was 100%.

Of the 24 patients included in the analyses, 10 (41.7%) were men and 14 (58.3%) were women, and the mean age ± standard deviation (SD) was 66.4 ± 12.0 years (Table 1). The rash was judged to be mild in seven cases (29.2%), moderate in 12 cases (50.0%), and severe in five cases (20.8%). Based on the distribution of the rash, case photographs, and the reports of the attending physicians, there were no cases of generalized herpes zoster. The time from herpes zoster onset to the start of treatment (mean ± SD) was 3.3 ± 1.1 days, and the most common sites of onset of herpes zoster were the thoracic back in nine patients (37.5%), the lumbar back and buttocks in seven patients (29.2%), and the abdominal back in six patients (25.0%). The most common comorbid condition was malignancy (12 cases [50.0%]), and the most common treatment methods for the comorbid condition were oral steroid therapy (12 cases [50.0%]) and anticancer chemotherapy (10 cases [41.7%]).

**TABLE 1:** Baseline characteristics (safety analysis population).

Background factor	Variable	Patients (*n* = 24)
Sex	Male	10 (41.7)
	Female	14 (58.3)
Age (years)	<50	1 (4.2)
	50–60	7 (29.2)
	60–69	5 (20.8)
	70–79	9 (37.5)
	≥80	2 (8.3)
		66.4 ± 12.0
Time from onset of herpes zoster to start of treatment (days)	2	8 (33.3)
	3	5 (20.8)
	≥4	11 (45.8)
		3.3 ± 1.1
Site of onset of herpes zoster^a^	Head	0 (0.0)
	Face	1 (4.2)
	Neck	2 (8.3)
	Arms	2 (8.3)
	Thoracic back	9 (37.5)
	Abdominal back	6 (25.0)
	Lumbar back and buttocks	7 (29.2)
	Legs	3 (12.5)
Varicella vaccination history	Having	1 (4.2)
	None	23 (95.8)
Condition causing immunosuppression	Malignant tumor	12 (50.0)
	Transplantation	0 (0.0)
	Collagen disease	3 (12.5)
	Other	9 (37.5)
Treatment of primary disease^a^	Chemotherapy	10 (41.7)
	Oral steroid	12 (50.0)
	Intravenous steroid	4 (16.7)
	Immunosuppressant drug	3 (12.5)
	JAK inhibitor	3 (12.5)
	Biologics	2 (8.3)
	Antifolate	1 (4.2)
	Radiation therapy	1 (4.2)

*Note*: Values are expressed as number (percentage) or mean ± standard deviation (SD).

Abbreviation: JAK, Janus kinase.

^a^ More than one could be selected.

From day 1 of treatment with amenamevir, 22 patients used a total of 58 pain medications as adjunctive therapy; this included 21 patients using a total of 40 medications for pain due to herpes zoster. Table S3 shows the breakdown of pain medications used for pain due to herpes zoster by drug.

### 3.2 Primary efficacy end point

**FIGURE 1:**
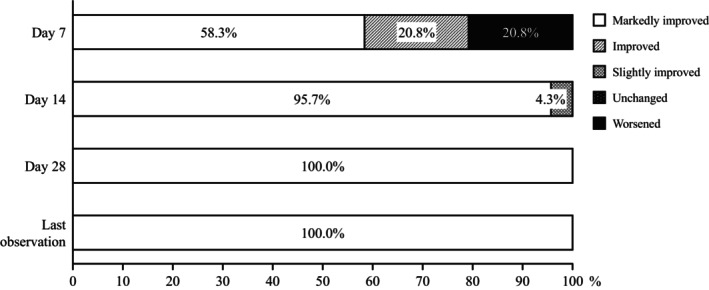
Overall improvement in skin symptoms (full analysis set). Improvement of skin symptoms was evaluated by a Central Evaluation Committee (Table S2) by comparing photographs of the affected area on days 1, 7, 14, and 28.

The overall improvement of skin symptoms on day 7 was markedly improved for 58.3% (14 of 24 patients), improved for 20.8% (five of 24 patients), and worsened for 20.8% (five of 24 patients). The combined improvement rate was 79.2% (95% CI, 57.8–92.9) (Figure 1).

### 3.3 Secondary efficacy end points

On day 14, 22 of 23 evaluable patients (95.7%) were markedly improved and one of 23 patients (4.3%) was slightly improved in terms of overall improvement of skin symptoms. On day 28, the overall improvement of skin symptoms was markedly improved in all patients who were evaluated.

**FIGURE 2:**
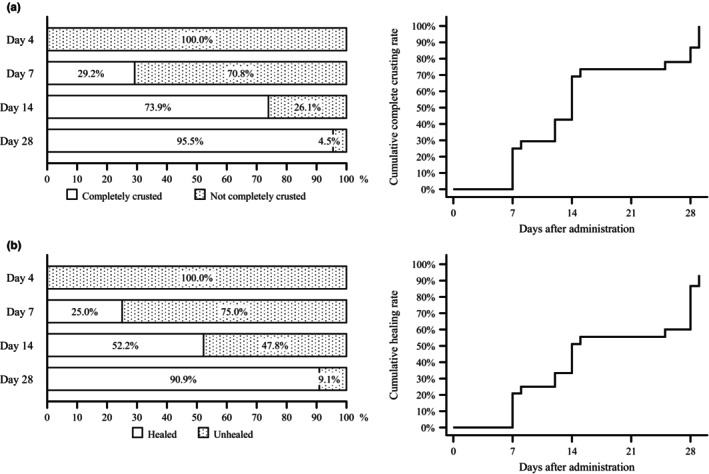
Crusting and healing (full analysis set). (a) Proportion of patients with complete crusting and cumulative complete crusting rate; (b) proportion of patients with healing and cumulative healing rate.

Regarding the transition of skin symptoms, it was shown that crusting progressed with treatment, peaking on day 7, and that skin symptoms then moved toward a normal (healed) state (Figure S3). The median times to complete crusting and to healing were both 14 days using the Kaplan–Meier method (Figure 2). Images of skin symptoms on days 1, 7, and 14 are shown in Figure S4, illustrating an example of a patient whose skin symptoms improved and an example of a patient whose skin symptoms did not improve on day 7 but on day 14.

**FIGURE 3:**
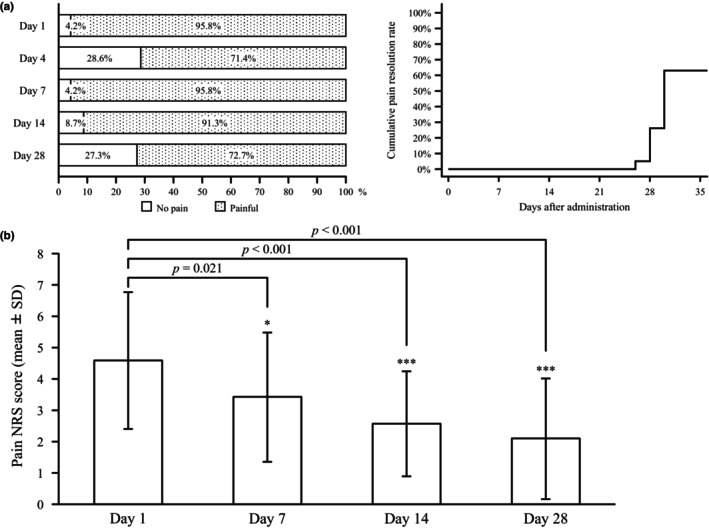
Evaluation of pain (full analysis set). (a) Proportion of patients with pain resolution and cumulative pain resolution rate; (b) pain numeric rating scale (NRS) score at each time point. SD, standard deviation.

The median time to pain resolution was 30 days after the start of treatment (Figure 3), and there was a significant difference in pain NRS scores between day 1 and each of days 7, 14, and 28 (*p* = 0.021, *p* < 0.001, and *p* < 0.001, respectively), with a trend toward a decrease over time. However, it should be noted that pain medications (Table S3) may have influenced the results.

**FIGURE 4:**
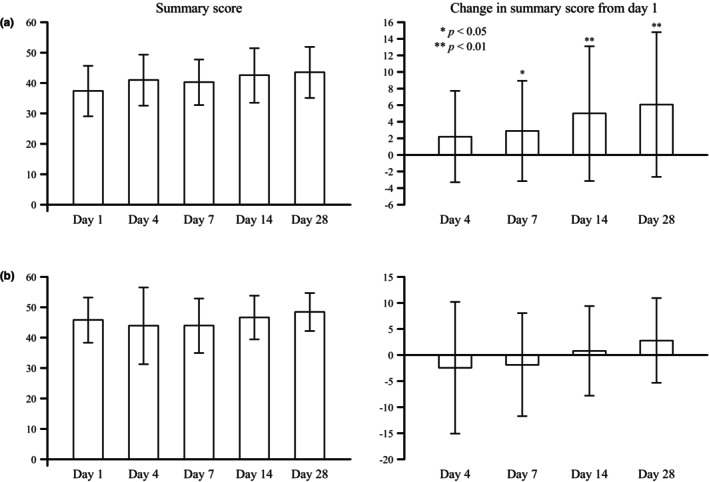
Quality of life (QOL) improvement (full analysis set). Summary scores and changes in summary scores from day 1 for (a) physical health and (b) mental health. QoL improvement was defined as the difference in the short‐form 8‐item health survey (SF‐8) score from day 1 at each timepoint.

Of the SF‐8 scores, improvement in the overall health perspective score was observed from day 7, and improvement in the vitality score was observed from day 14. Improvements in physical pain and daily role functioning (mental) scores were also observed on day 28 (data not shown). The overall summary score for physical health showed improvement from day 7, while the summary score for mental health showed no change (Figure 4).

### 3.4 VZV‐specific antibody titer

**FIGURE 5:**
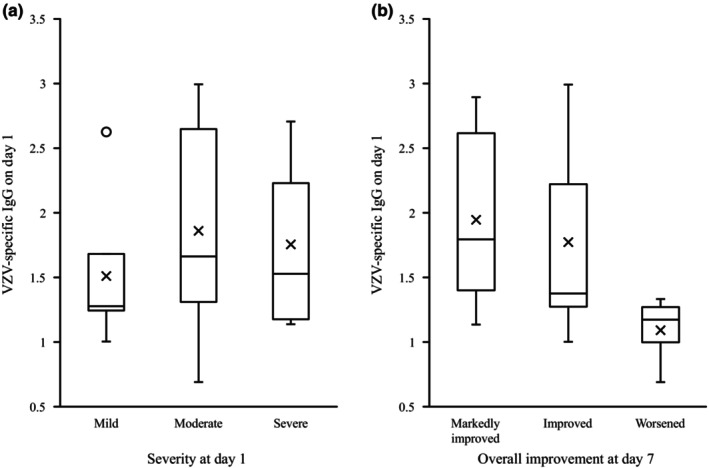
Relationship between varicella zoster virus (VZV)–specific antibody titers and symptoms. (a) VZV‐specific IgG antibody titers on day 1 relative to rash severity on day 1; (b) relationship between VZV‐specific IgG antibody titer on day 1 and the degree of general improvement of skin symptoms on day 7.

All 24 patients had positive VZV‐specific IgG antibody titers on day 1 (Table 2). In the five patients whose overall improvement worsened on day 7, VZV‐specific IgG antibody titers were very low (4.9–21.2) in all cases. Further, when an IgG antibody titer <50 was defined as low level,^10^ 15 patients were judged to have low IgG antibody titers (Table 3). In addition, five patients (20.8%) had positive VZV‐specific IgM antibody titers on day 1 (Table 2). Although there was no clear association between VZV‐specific IgG antibody titer on day 1 and rash severity (Figure 5a), a higher VZV‐specific IgG antibody titer on day 1 was associated with better overall improvement of skin symptoms on day 7 (Figure 5b).

**TABLE 2:** Distribution of VZV‐specific antibody titers on day 1.

IgG	Number	Percentage	IgM	Number	Percentage
<2 (negative)	0	0.0	<0.8 (negative)	19	79.2
≥2 to <4	0	0.0	≥0.8 to <1.2	0	0.0
≥4 to <50	15	62.5	≥1.2	5	20.8
≥50 to <100	1	4.2			
≥100	8	33.3			
Total	24	100.0	Total	24	100.0

Abbreviation: VZV, varicella zoster virus.

**TABLE 3:** Listing of VZV‐specific antibody titers.

ID	IgG		IgM		Severity at baseline	Overall improvement on day 7
	Day 1	Day 7	Day 1	Day 7		
01–001	133	1020	**1.57**	2.12	Moderate	Markedly improved
01–002	25.4	310	0.45	0.21	Moderate	Markedly improved
01–003	33.7	3020	0.38	0.98	Severe	Markedly improved
01–004	19.0	171	0.33	0.43	Mild	Improved
03–001	988	500	**3.94**	2.83	Moderate	Improved
03–002	792	932	**4.16**	4.28	Moderate	Markedly improved
03–003	83.0	220	0.25	0.47	Moderate	Markedly improved
03–004	424	1560	**4.36**	5.75	Mild	Markedly improved
03–005	396	299	0.39	0.45	Moderate	Markedly improved
03–006	24.0	152	0.26	0.53	Moderate	Improved
05–001	19.6	485	0.33	1.20	Moderate	Markedly improved
05–002	13.7	836	0.15	5.48	Severe	Markedly improved
07–001	511	353	**7.30**	7.56	Severe	Markedly improved
08–001	17.6	146	0.39	0.30	Mild	Markedly improved
08–002	48.1	158	0.64	2.27	Mild	Markedly improved
08–003	29.1	74.0	0.37	0.31	Mild	Markedly improved
08–004	**21.2**	1000	0.34	0.46	Moderate	Worsened
08–005	**10.0**	571	0.07	1.61	Moderate	Worsened
09–001	500	240	0.54	0.53	Moderate	Markedly improved
09–002	10.1	55.1	0.34	7.08	Mild	Improved
09–003	**4.9**	1100	0.46	6.04	Moderate	Worsened
10–001	**18.8**	518	0.36	1.26	Mild	Worsened
10–002	**15.0**	13.1	0.24	0.29	Severe	Worsened
10–003	170	589	0.79	1.03	Severe	Improved

*Note*: Bold letters in IgG antibody titers on day 1 indicate cases (*n* = 5) in which the overall improvement was judged to be “worsened” on day 7. Bold letters in IgM antibody titers on day 1 indicate varicella zoster virus (VZV)–specific IgM antibody titer‐positive (≥0.8) cases (*n* = 5).

### 3.5 Safety

A total of 22 AEs were observed in 11 of 24 patients; this included two events of hyponatremia (two of 24 patients [8.3%]) (Table 4). All other AEs occurred in one patient each (4.2%). No clinically relevant changes in laboratory values were observed.

**TABLE 4:** AEs (safety analysis population).

	No. of patients (%)	No. of events
All	11 (45.8)	22
Infections and infestations	4 (16.7)	4
Impetigo	1 (4.2)	1
Nasopharyngitis	1 (4.2)	1
Skin bacterial infection	1 (4.2)	1
Acarodermatitis	1 (4.2)	1
Neoplasms benign, malignant and unspecified (including cysts and polyps)	1 (4.2)	1
Cancer pain	1 (4.2)	1
Blood and lymphatic system disorders	1 (4.2)	1
Anemia	1 (4.2)	1
Immune system disorders	1 (4.2)	1
Hypogammaglobulinemia	1 (4.2)	1
Metabolism and nutrition disorders	2 (8.3)	3
Hyponatremia	2 (8.3)	2
Hyperkalemia	1 (4.2)	1
Psychiatric disorders	1 (4.2)	1
Insomnia	1 (4.2)	1
Nervous system disorders	2 (8.3)	2
Dizziness	1 (4.2)	1
Headache	1 (4.2)	1
Gastrointestinal disorders	1 (4.2)	1
Nausea	1 (4.2)	1
Hepatobiliary disorders	1 (4.2)	1
Hepatic function abnormal	1 (4.2)	1
Skin and subcutaneous tissue disorders	3 (12.5)	3
Erythema multiforme	1 (4.2)	1
Keloid scar	1 (4.2)	1
Purpura	1 (4.2)	1
Renal and urinary tract disorders	2 (8.3)	2
Dysuria	1 (4.2)	1
Renal impairment	1 (4.2)	1
General disorders and administration site conditions	1 (4.2)	1
Pyrexia	1 (4.2)	1
Investigations	1 (4.2)	1
Blood pressure decreased	1 (4.2)	1

*Note*: Adverse events (AEs) are shown according to the System Organ Class and Preferred Terms of the *Medical Dictionary for Regulatory Activities*, Japanese version 23.0.

Five AEs (bacterial skin infection, anemia, hyponatremia, headache, and liver function abnormality) with a possible causal relationship to amenamevir were observed in one patient; all were reported to be either recovered or recovering. There were two serious AEs (bacterial skin infection and cancer pain; *n* = 1 each), and a causal relationship with amenamevir could not be ruled out for the bacterial skin infection.

Thrombocytopenia, cardiovascular events, and renal impairment were identified as potential risks in the drug risk management plan for amenamevir. One case of renal dysfunction was reported, but a causal relationship was denied by the investigator because it was not serious and occurred 19 days after completion of amenamevir treatment.

## 4 DISCUSSION

This study examined the efficacy and safety of amenamevir in patients who developed herpes zoster while receiving immunosuppressive drugs primarily to treat moderate to severe rashes. We found that 19 of 24 patients (79.2%) showed improvement (58.3% markedly improved and 20.8% improved) in overall skin symptoms on day 7.

Five patients had prolonged or temporarily worsening symptoms, but all recovered. We found no clinical signs that could predict a worsening or prolonged course at the initial visit, and the results for both skin symptoms and the number of days to complete crusting and days to healing indicated that herpes zoster recovered (healed) over time in these patients.

It has been reported that the maximum plasma drug concentration and area under the curve of amenamevir when administered under fasting conditions were approximately 0.64‐ and 0.52‐fold, respectively, of those when amenamevir was administered postprandially.^11^ In patients for whom sufficient efficacy was not observed on day 7, the overall quantity and nutritional content of meals taken during the amenamevir treatment period might have been insufficient, resulting in suboptimal absorption of amenamevir. In addition, amenamevir is metabolized by CYP3A and has been shown to induce both CYP3A and CYP2B6,^12^ and we can postulate that polypharmacy in the treatment of malignancies and autoimmune diseases may have affected the efficacy of amenamevir, as well as the underlying condition. Thus, background factors, including feeding status, should be noted when using amenamevir in immunosuppressed patients.

It is generally known that patients taking Janus kinase (JAK) inhibitors have a higher risk of developing herpes zoster and may also have more severe and prolonged symptoms.^13^ JAK inhibitors were used by two patients in this study. One had severe rash on day 1, and the JAK inhibitor was discontinued during amenamevir treatment and resumed on day 7 when herpes zoster had improved. The second patient continued to receive a JAK inhibitor and methotrexate throughout amenamevir treatment; in this patient, although the rash was mild on day 1, it worsened by day 7. It is recommended that patients taking JAK inhibitors are made aware of the risks of developing herpes zoster and severe symptoms, and that treatment with an antiherpesvirus agent such as amenamevir should be started as soon as possible after the onset of herpes zoster symptoms; a short‐term withdrawal of JAK inhibitors should also be considered.

Acute pain and postherpetic neuralgia in herpes zoster are collectively referred to as zoster‐associated pain (ZAP), and ZAP is known to reduce patients' QOL levels and have a significant impact on their physical and mental health.^14^ Early administration of antiherpesvirus drugs can suppress viral replication, resulting in early improvement of the rash and also shortening the time until ZAP disappears.^15^ In the current study, pain NRS scores decreased over time after treatment initiation, and SF‐8 QOL scores, including the summary score for physical health, improved over time after treatment initiation.

In the current study, we also examined the relationship between VZV‐specific antibody titers and the efficacy of amenamevir. Five patients (20.8%) were positive for VZV‐specific IgM antibody titers on day 1, a slightly higher frequency than that of the general population of patients with herpes zoster seen in routine clinical practice.^16^ Of the 19 patients who were negative for VZV‐specific IgM antibody titers on day 1, eight (33.3%) became positive by day 7. For VZV‐specific IgG antibody titers, a value <50 was defined as a low IgG value^10^; based on this threshold, 15 of 24 patients had low VZV‐specific IgG titers on day 1, of whom seven had titers >50 by day 7. It is considered that these low VZV‐specific antibody titers and sluggish elevation rates may have been due to immunosuppressive treatments against the primary disease. Specifically, immune suppression may have decreased cellular immunity, as reflected by the antibody titer. Moreover, the situation mimics the status during a primary infection, when the host lacks immunity to VZV; thus, the shortage of existing neutralizing antibodies may have induced new B‐cell clones to produce VZV‐specific IgM antibodies in these patients.

There was no clear relationship between VZV‐specific IgG antibody titer and rash severity on day 1. However, very low VZV‐specific IgG antibody titer on day 1 may be related to the worsening observed on day 7. The five patients who were classified as “worsened” in terms of general improvement all had very low VZV‐specific IgG antibody titers (4.9–21.2) on day 1, suggesting that their skin symptoms may have been prolonged or temporarily worsened despite amenamevir administration, due to insufficient immune response. This novel finding, that a very low titer of VZV‐specific IgG antibody at the beginning of herpes zoster may reduce the efficacy of antiviral treatment, has not been previously reported. However, it is not practical to routinely measure VZV‐specific IgG antibody titers in clinical practice. Since 20.8% of patients in this study had worsened skin symptoms on day 7, more frequent observation is recommended, and clinicians should consider switching to an alternative therapy such as intravenous acyclovir in immunosuppressed patients with herpes zoster who fail to respond to amenamevir.

This study included a group of patients with medical backgrounds such as malignancy or autoimmune disease that should be considered as important safety factors, compared with the general population of patients with herpes zoster. Five AEs (bacterial skin infection, anemia, hyponatremia, headache, and abnormal liver function) in one patient may have had a causal relationship to amenamevir, but none required discontinuation of amenamevir treatment and the outcomes were recovered or recovering. This patient was presumed to have been susceptible to infection due to the effects of preexisting diabetes, in addition to immunosuppression associated with treatment for pulmonary and esophageal carcinoma. Therefore, underlying medical conditions and treatment should also be considered when considering the use of amenamevir in immunosuppressed patients.

When this study was compared with the interim analysis results of an earlier postmarketing surveillance,^17^ our study population was found to have slightly higher rash severity, pain level, and age at baseline, due to the underlying condition and its treatment. However, in terms of skin symptoms, there seemed to be no difference between the studies in the number of days to resolution of erythema and papules, blisters and pustules, or complete crusting, nor was there a large discrepancy in the number of days to resolution of pain between the two study populations. Taken together, these data suggest similar efficacy and safety in patients with compromised immune function compared with the general population with herpes zoster.

Due to the low permeability of amenamevir into the central nervous system (CNS), which limits its therapeutic efficacy against CNS infection, and the subsequent risk of zoster‐related meningitis or meningoencephalitis,^18^, ^19^, ^20^, ^21^ consideration should be given to administering alternative treatment (such as intravenous acyclovir) to immunosuppressed patients with lesions in the area of the trigeminal nerve and at high risk of CNS VZV infection.

The main limitation of the current study was the small study size, which was affected by the COVID‐19 epidemic, resulting in limited enrollment. Moreover, the diversity of patient background factors, including their underlying conditions (a range of malignant tumors and autoimmune diseases) and treatments, makes it difficult to draw widely generalizable conclusions. Thus, future studies with a larger number of patients are needed. However, this exploratory study has demonstrated that it is possible to treat immunosuppressed patients who have herpes zoster. Amenamevir could be used effectively and safely in this patient population, although physicians must carefully observe the course of symptoms, take into account patient background factors such as the content of treatment for the underlying disease, and monitor the amount of food eaten before taking amenamevir.

## FUNDING INFORMATION

This study was funded by Maruho Co., Ltd.

## CONFLICT OF INTEREST STATEMENT

This study was funded by Maruho Co., Ltd. S. I. and T. Y. received speaker fees, research funding, and fees for arranging education from Maruho Co., Ltd. M.A. and D.O. received fees for arranging education from Maruho Co., Ltd. S. S. received speaker fees from Maruho Co., Ltd. S.Y. received speaker fees and research funding from Maruho Co., Ltd. H.F. is an employee of Maruho Co., Ltd. All other authors report that they have no conflicts of interest to declare. S.I. is an editorial board member of the *Journal of Dermatology*. To minimize bias, S.I. was excluded from all editorial decision‐making related to the acceptance of this article for publication.

## Supporting information


Figure S1.

Table S1.

## References

[1] Gnann JW Jr , Whitley RJ . Clinical practice Herpes zoster. N Engl J Med. 2002;347:340–346. 12151472 10.1056/NEJMcp013211

[2] Oxman MN . Zoster vaccine: current status and future prospects. Clin Infect Dis. 2010;51:197–213. 20550454 10.1086/653605

[3] Cohen JI . Clinical practice: herpes zoster. N Engl J Med. 2013;369:255–263. 23863052 10.1056/NEJMcp1302674 PMC4789101

[4] Arvin AM , Gilden D . Varicella‐zoster virus. In: Knipe DM , Howley PM , editors. Fields virology. Philadelphia: Lippincott Williams & Wilkins; 2013. p. 2015–263.

[5] Harpaz R , Ortega‐Sanchez IR , Seward JF . Prevention of herpes zoster: recommendations of the advisory committee on immunization practices (ACIP). MMWR Recomm Rep. 2008;57:1–30. 18528318

[6] Smitten AL , Choi HK , Hochberg MC , Suissa S , Simon TA , Testa MA , et al. The risk of herpes zoster in patients with rheumatoid arthritis in the United States and the United Kingdom. Arthritis Rheum. 2007;57:1431–1438. 18050184 10.1002/art.23112

[7] Chen SY , Suaya JA , Li Q , Galindo CM , Misurski D , Burstin S , et al. Incidence of herpes zoster in patients with altered immune function. Infection. 2014;42:325–334. 24214127 10.1007/s15010-013-0550-8 PMC3968442

[8] Chono K , Katsumata K , Kontani T , Kobayashi M , Sudo K , Yokota T , et al. ASP2151, a novel helicase‐primase inhibitor, possesses antiviral activity against varicella‐zoster virus and herpes simplex virus types1 and 2. J Antimicrob Chemother. 2010;65:1733–1741. 20534624 10.1093/jac/dkq198

[9] Kusawake T , Kowalski D , Takada A , Kato K , Katashima M , Keirns JJ , et al. The influence of hepatic and renal impairment on the pharmacokinetics of a treatment for herpes zoster, amenamevir (ASP2151): phase 1, open‐label, single‐dose, parallel‐group studies. Adv Ther. 2017;34:2612–2624. 29134428 10.1007/s12325-017-0643-3 PMC5709452

[10] Yamakawa K , Hamada M , Takeda T . Assessment of anti‐VZV IgG antibodies in patients with facial palsy. Comparison between CF, FA and EIA. Facial N Res Jpn. 2004;24:53–56.

[11] Kusawake T , Keirns JJ , Kowalski D , den Adel M , Groenendaal‐van de Meent D , Takada A , et al. Pharmacokinetics and safety of amenamevir in healthy subjects: analysis of four randomized phase 1 studies. Adv Ther. 2017;34:2625–2637. 29134426 10.1007/s12325-017-0642-4 PMC5709458

[12] Maeda H , Nakamura H , Kikukawa Y . Pharmacological profiles and clinical effects of amenamevir tablet as treatments for herpes zoster. Folia Pharmacol Jpn. 2019;153:35–43. 10.1254/fpj.153.35 30643090

[13] Sunzini F , McInnes I , Siebert S . JAK inhibitors and infections risk: focus on herpes zoster. Ther Adv Musculoskelet Dis. 2020;12:1759720X20936059. 10.1177/1759720X20936059 PMC7328488 32655703

[14] Johnson RW , Bouhassira D , Kassianos G , Leplège A , Schmader KE , Weinke T . The impact of herpes zoster and post‐herpetic neuralgia on quality‐of‐life. BMC Med. 2010;8:37. 20565946 10.1186/1741-7015-8-37 PMC2905321

[15] Tyring S , Barbarash RA , Nahlik JE , Cunningham A , Marley J , Heng M , et al. Famciclovir for the treatment of acute herpes zoster: effects on acute disease and postherpetic neuralgia. A randomized, double‐blind, placebo‐controlled trial. Collaborative Famciclovir Herpes Zoster Study Group. Ann Intern Med. 1995;123:89–96. 7778840 10.7326/0003-4819-123-2-199507150-00002

[16] Imafuku S . Herpes zoster and subunit vaccine. Virus. 2021;71:45–54. 35526994 10.2222/jsv.71.45

[17] Imafuku S , Korematsu K , Mori N , Kani T , Matsui K . Investigation of the safety and efficacy of amenamevir (Amenalief® tablet 200 mg) in patients with herpes zoster (interim report from a special drug use‐result survey). J Jpn Org Clin Dermatol. 2020;37:641–649.

[18] Omiya M , Nishiguchi S , Moriya H , Akazawa K , Nagahiro T , Seto M . Aseptic meningitis after amenamevir treatment for herpes zoster ophthalmicus with oculomotor nerve palsy in a patient taking immunosuppressant. J Infect Chemother. 2023;29:519–522. 36708771 10.1016/j.jiac.2022.12.008

[19] Itoh K , Mitsuke Y , Wakahara M , Yoshioka T , Otsuki N , Suzuki Y , et al. Aseptic meningitis after amenamevir treatment for herpes zoster in the first branch of the trigeminal nerve. Intern Med. 2022;61:2809–2811. 35228415 10.2169/internalmedicine.8581-21 PMC9556230

[20] Tada S , Kaito Y , Watanabe A , Sugiyama Y , Nishigaichi A , Miwa T , et al. Varicella‐zoster meningitis and myelitis after herpes zoster dermatitis treatment with amenamevir: a case series and literature review. Cureus. 2024;16:e54775. 38524092 10.7759/cureus.54775 PMC10961168

[21] Taniguchi Y , Kano Y , Kitamura T , Miura T , Yamada K . Varicella‐zoster meningoencephalitis and vasculitis after treatment with amenamevir to herpes zoster in the trigeminal nerve area. Rinsho Shinkeigaku. 2021;61:239–242. 33762495 10.5692/clinicalneurol.cn-001531

