# Supplementary material for: An exploratory study of the efficacy and safety of amenamevir for the treatment of herpes zoster in patients receiving immunosuppressive drugs
Source: J Dermatol. 2024 Jul 24;51(10):1279–89. doi: 10.1111/1346-8138.17364 (PMC11483900; doi:10.1111/1346-8138.17364)
Supplement: Supplementary file 1 — Figure S1. [file JDE-51--s002.pptx]

## Slide 1
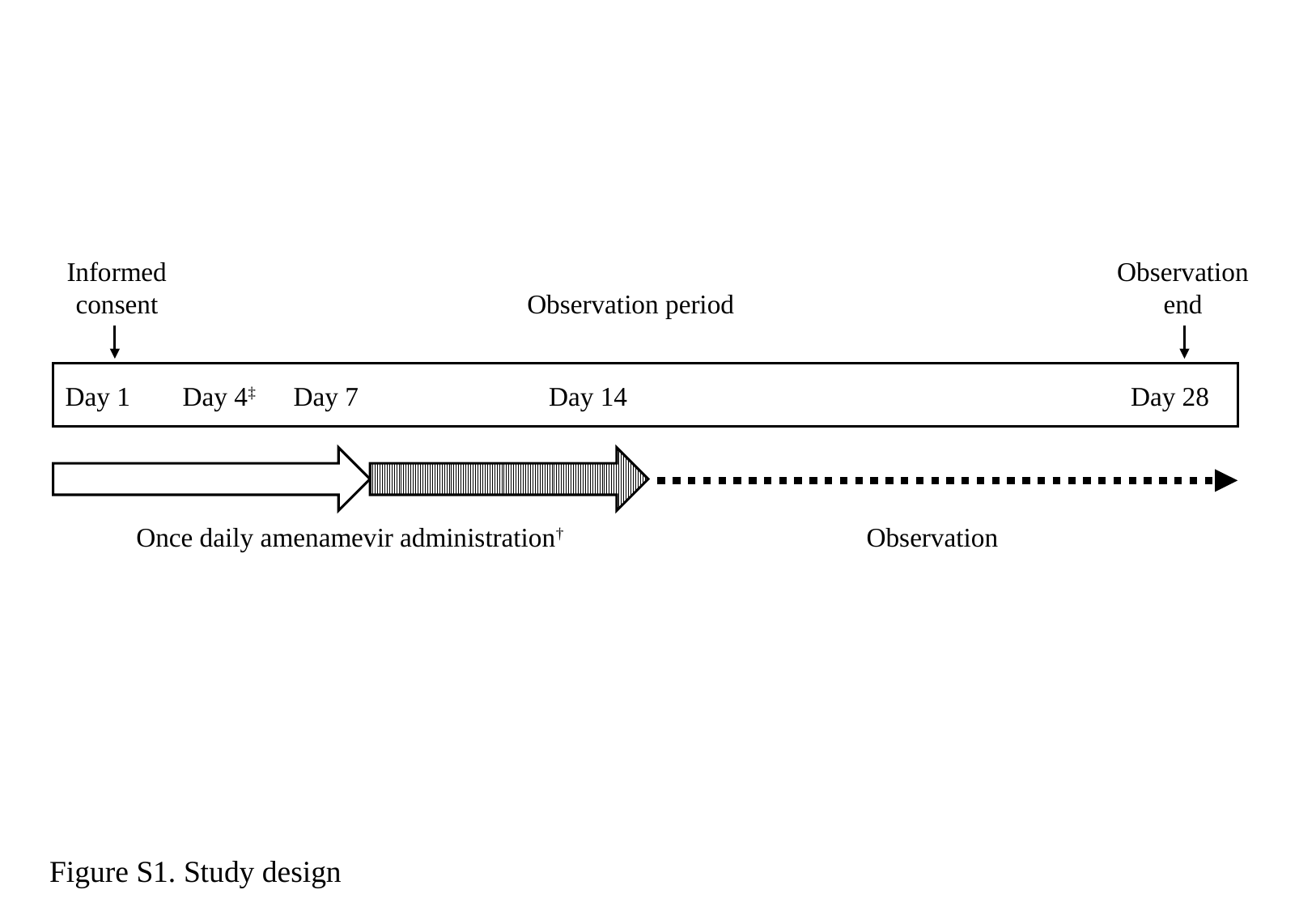

Informed consent
Observation
end
Observation period
Day 1
Day 4‡
Day 7
Day 14
Day 28
Once daily amenamevir administration†
Observation
Figure S1. Study design

## Slide 2
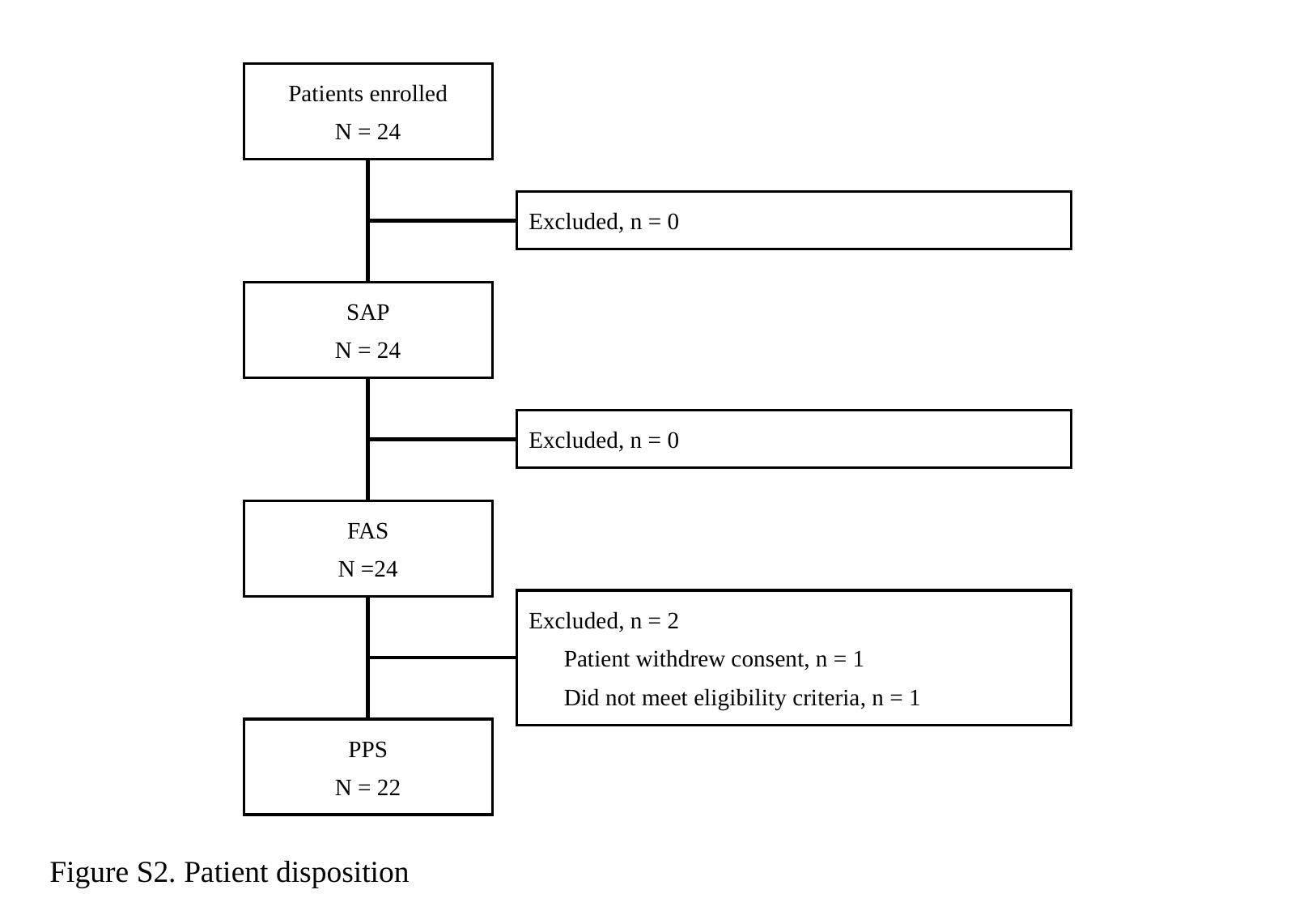

Patients enrolled
N = 24
Excluded, n = 0
SAP
N = 24
Excluded, n = 0
FAS
N =24
Excluded, n = 2
 Patient withdrew consent, n = 1
 Did not meet eligibility criteria, n = 1
PPS
N = 22
Figure S2. Patient disposition

## Slide 3
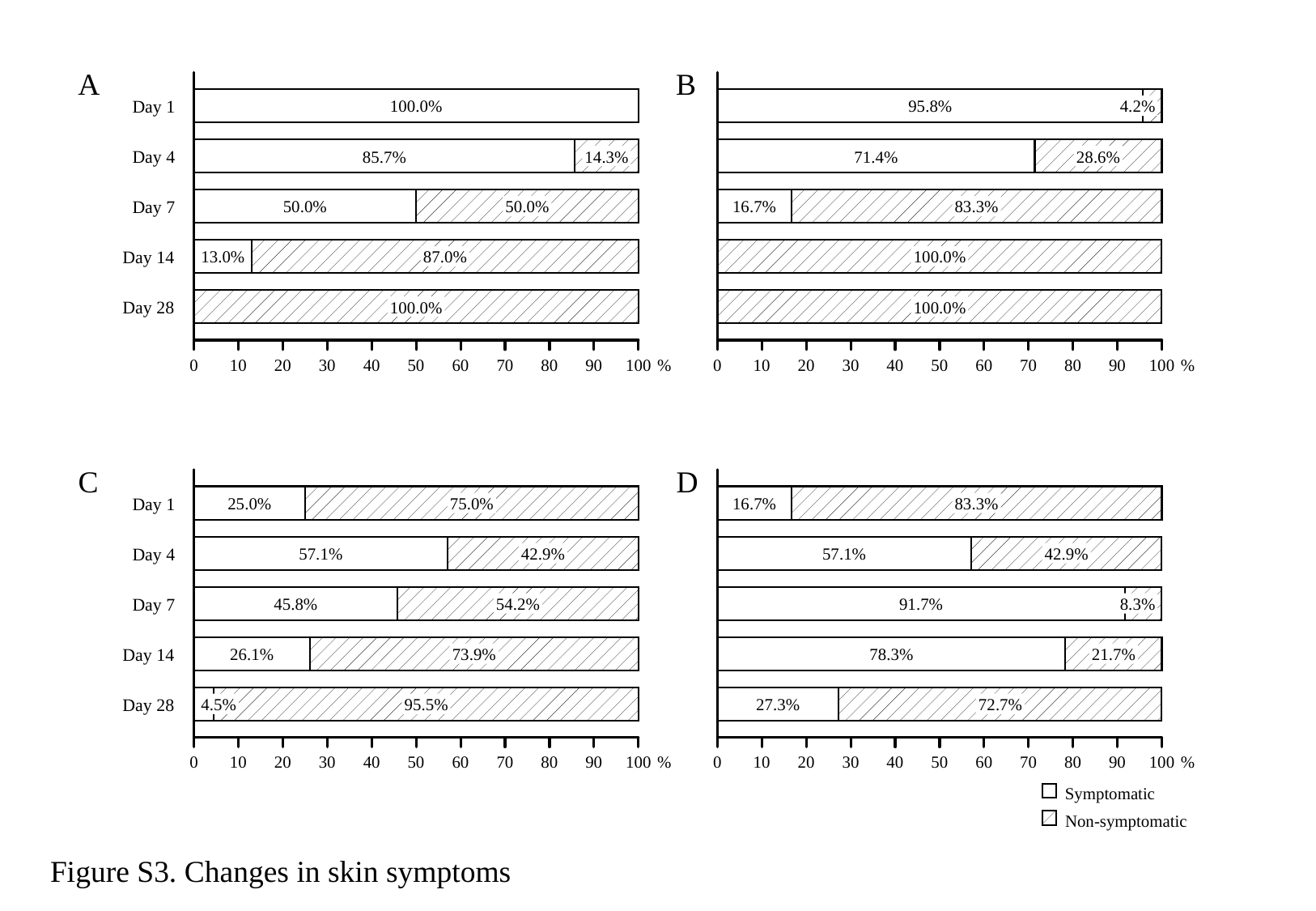

A
B
Day 1
100.0%
Day 4
85.7%
14.3%
Day 7
50.0%
50.0%
Day 14
13.0%
87.0%
Day 28
100.0%
0
10
20
30
40
50
60
70
80
90
100
%
95.8%
4.2%
71.4%
28.6%
16.7%
83.3%
100.0%
100.0%
0
10
20
30
40
50
60
70
80
90
100
%
C
D
Day 1
25.0%
75.0%
Day 4
57.1%
42.9%
Day 7
45.8%
54.2%
Day 14
26.1%
73.9%
Day 28
4.5%
95.5%
0
10
20
30
40
50
60
70
80
90
100
%
16.7%
83.3%
57.1%
42.9%
91.7%
8.3%
78.3%
21.7%
27.3%
72.7%
0
10
20
30
40
50
60
70
80
90
100
%
Symptomatic
Non-symptomatic
Figure S3. Changes in skin symptoms

## Slide 4
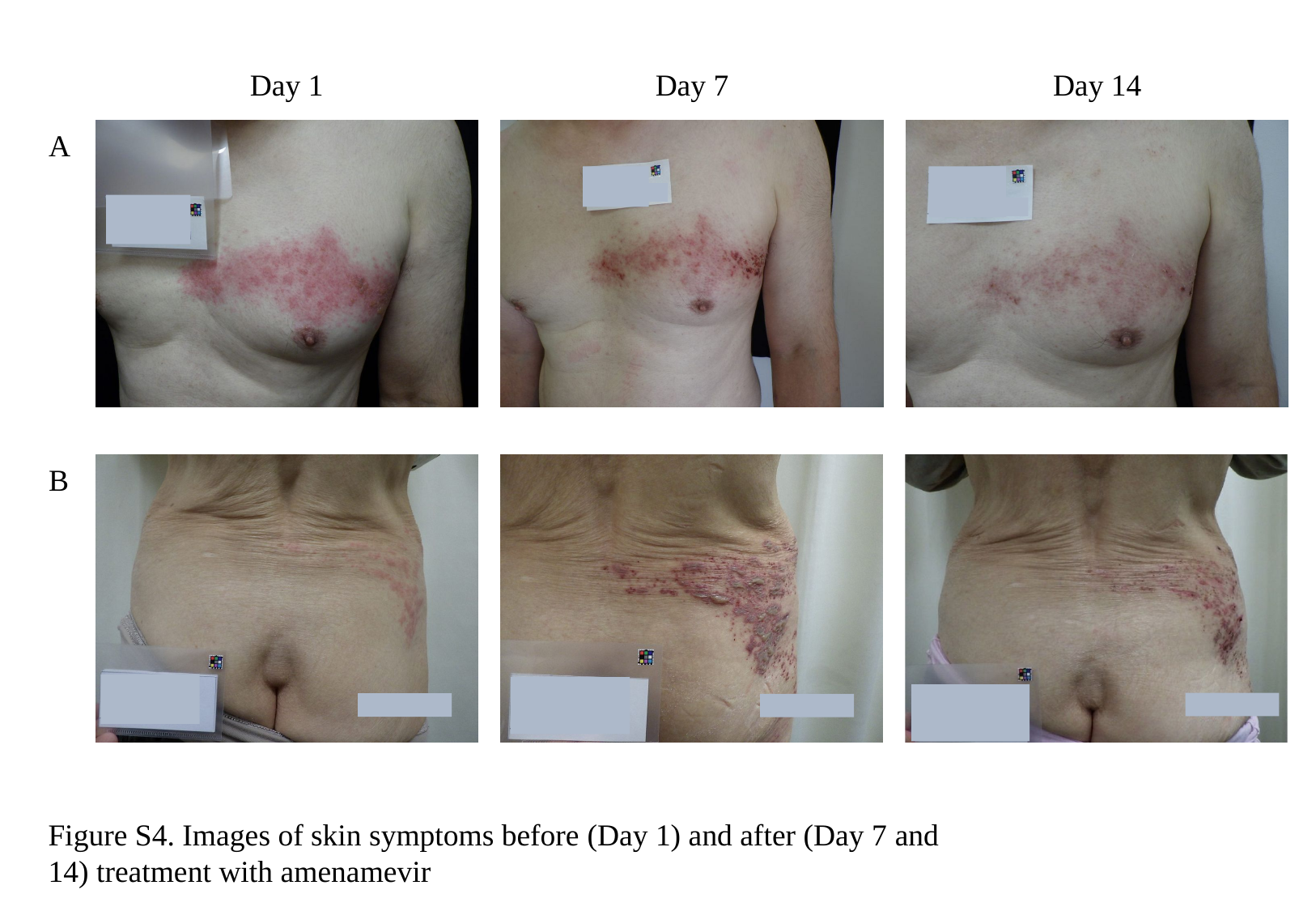

Day 1
Day 7
Day 14
A
B
Figure S4. Images of skin symptoms before (Day 1) and after (Day 7 and 14) treatment with amenamevir
