# Supplementary material for: An exploratory study of the efficacy and safety of amenamevir for the treatment of herpes zoster in patients receiving immunosuppressive drugs
Source: J Dermatol. 2024 Jul 24;51(10):1279–89. doi: 10.1111/1346-8138.17364 (PMC11483900; doi:10.1111/1346-8138.17364)
Supplement: Supplementary file 2 — Table S1. [file JDE-51--s001.docx]

**Supporting Information**

Figure S1. Study design

^†^Amenamevir was to be administered for 7 days. However, administration could be continued up to Day 14 at the discretion of the investigator, according to the patient’s skin symptoms.

^‡^Evaluations were to be completed on Day 4 if feasible.

Figure S2. Patient disposition

FAS, full analysis set; PPS, per protocol set; SAP, safety analysis set.

Figure S3. Changes in skin symptoms (full analysis set). A) Erythema and papules; B) blisters and pustules; C) erosions and ulcers; D) crusts

Figure S4. Images of skin symptoms before (Day 1) and after (Day 7 and Day 14) treatment with amenamevir. A) Example of a patient with improved skin symptoms (front view); B) example of a patient without improved skin symptoms (back view)

Table S1. List of participating institutions and principal investigators

|  | Institutions | Address | Principal investigators |
| --- | --- | --- | --- |
| 1 | Department of Dermatology, and Cosmetic Surgery, Fukuoka University Hospital | 7-45-1 Nanakuma, Jonan-ku, Fukuoka City, Fukuoka, Japan | Shinichi Imafuku |
| 2 | Department of Dermatology, Federation of National Public Service Personnel Mutual Aid Associations, Hamanomachi Hospital | 3-3-1 Nagahama, Chuo-ku, Fukuoka City, Fukuoka, Japan | Satoshi Takeuchi |
| 3 | Department of Dermatology and Allergology, National Hospital Organization Kyushu Medical Center | 1-8-1 Jigyohama, Chuo-ku, Fukuoka City, Fukuoka, Japan | Kazunori Urabe |
| 4 | Department of Dermatology, Kurume University Hospital | 67 Asahimachi, Kurume City, Fukuoka, Japan | Masataka Arakawa (-Feb. 15, 2022)  Eri Katayama (Feb. 16, 2022-) |
| 5 | Department of Dermatology, Hiroshima Red Cross Hospital and Atomic-bomb Survivors Hospital | 1-9-6 Sendamachi, Naka-ku, Hiroshima City, Hiroshima, Japan | Ryo Sasaki |
| 6 | Department of Dermatology, Kawasaki Medical School Hospital | 577 Matsushima, Kurashiki City, Okayama, Japan | Daigo Oka (-Feb. 15, 2022)  Rehito Mashiko (Feb. 16, 2022-) |
| 7 | Department of Dermatology, Kawasaki Medical School General Medical Center | 2-6-1 Nakasange, Kita-ku, Okayama City, Okayama, Japan | Takenobu Yamamoto |

Table S2. Members of the Central Evaluation Committee

|  | Affiliation | Address | Name |
| --- | --- | --- | --- |
| 1 | Sasori Dermatology Clinic | 1-15 Tsujiido-cho, Seki City, Gifu, Japan | Fumitake Ono |
| 2 | Department of Dermatology, Seirei Mikatahara General Hospital | 3453 Mikatahara-cho, Kita-ku, Hamamatsu City, Shizuoka, Japan | Shigeho Shirahama |
| 3 | Yasumoto Dermatology Clinic | 2-4-1 Harisuri Chuo, Chikushino City, Fukuoka, Japan | Shinichiro Yasumoto |

Table S3. Use of pain medications for pain due to herpes zoster (aggregated by drug)

| Drug name | Number of cases |
| --- | --- |
| Oral medication |  |
| Acetaminophen | 16 |
| Tramadol hydrochloride (including acetaminophen combination tablet) | 2 |
| Pregabalin | 13 |
| Mirogabalin besylate | 1 |
| Loxoprofen sodium hydrate | 5 |
| Celecoxib | 1 |
| Carbamazepine | 1 |
| Injection |  |
| Acetaminophen | 1 |
| Total | 40 |
